# Inversion evolutionary rates might limit the experimental identification of inversion breakpoints in non-model species

**DOI:** 10.1038/s41598-017-17650-1

**Published:** 2017-12-08

**Authors:** Eva Puerma, Dorcas J. Orengo, Montserrat Aguadé

**Affiliations:** Departament de Genètica, Microbiologia i Estadística, Facultat de Biologia and Institut de Recerca de la Biodiversitat (IRBio), Universitat de Barcelona, Barcelona, Spain

## Abstract

Chromosomal inversions are structural changes that alter gene order but generally not gene content in the affected region. In Drosophila, extensive cytological studies revealed the widespread character of inversion polymorphism, with evidence for its adaptive character. In *Drosophila subobscura*, polymorphism affects both its four large autosomal elements and its X (A) chromosome. The characterization of eight of these autosomal inversions breakpoints revealed that most of them originated through the staggered-breaks mechanism. Here, we have performed chromosomal walks to identify the breakpoints of two X-chromosome widely distributed inversions —A_2_ and A_1_— of *D*. *subobscura*. Inversion A_2_ is considered a warm-adapted arrangement that exhibits parallel latitudinal clines in the species ancestral distribution area and in both American subcontinents, whereas inversion A_1_ is only present in the Palearctic region where it presents an east-west cline. The duplication detected at the A_2_ inversion breakpoints is consistent with its origin by the staggered-breaks mechanism. Inversion A_1_ breakpoints could not be molecularly identified even though they could be narrowly delimited. This result points to chromosome walking limitations when using as a guide the genome of other species. Limitations stem from the rate of evolution by paracentric inversions, which in Drosophila is highest for the X chromosome.

## Introduction

Chromosomal inversions and gene duplications are two types of structural variation that have greatly contributed to genome evolution and organismal adaptation. Gene duplications are generally viewed as the major sources of evolutionary innovation by means of gene copy number change and subsequent adaptive change, whereas chromosomal inversions are considered the major contributors to genome reorganization through time as they alter the order of genes in the affected region but generally not its gene content. This view stems from interspecific comparisons based initially on sets of genes or markers, and more recently on whole genomes, and it refers therefore to fixed structural changes. In species of the Drosophila genus as well as in those of other dipteran genera with polytene chromosomes, surveys of chromosomal variation revealed the widespread character of chromosomal polymorphism due to paracentric inversions. Moreover, the concordant changes detected between the frequencies of some chromosomal arrangements and environmental variables (*e*.*g*., temperature in *Drosophila melanogaster* and *Drosophila subobscura*, and aridity in *Anopheles gambiae*) would support their adaptive character^1–6^.

The molecular characterization of the breakpoints of diverse polymorphic inversions as well as of recently fixed inversions revealed two major mechanisms originating inversions: i) ectopic recombination between inverted copies of a repetitive sequence^7^, and ii) staggered double-strand breaks and their subsequent repair, which leads to the presence of duplicated fragments in the inverted chromosome^8^. An inversion adaptive character is majorly attributed to its allelic content, which is preserved by the reduced recombination occurring in inversion heterokaryotypes. However, it can also be due to the putative functional effect of the structural change itself. Indeed, the inversion might change the regulatory environment of genes neighboring its breakpoints^9^. Moreover, in those cases where the inversion originated by the staggered-breaks mechanism, the resulting duplications might have dosage or chimeric effects^10,11^.

Both computational and experimental methodologies have been used to identify and molecularly characterize the breakpoints of polymorphic inversions in Drosophila. The first approach that requires genome-wide data of at least two individuals carrying alternative chromosomal arrangements (with and without a particular inversion) was developed and subsequently applied to identify and later characterize the breakpoints of eight polymorphic inversions of *D*. *melanogaster* (six cosmopolitan autosomal inversions and two rare endemic to Africa inversions of the X chromosome^12,13^). The breakpoints characterization revealed that five of the eight inversions had originated through the staggered-breaks mechanism, with the duplicated fragment ranging in size from 202 bp to 27 kb. The second approach is more laborious as it requires extensive experimental work that in its initial applications in *D*. *melanogaster* included either microdissection of polytene chromosome bands, library screening and *in situ* hybridization^14^, or the use of previously mapped P1 clones as probes for *in situ* hybridization on polytene chromosomes^15^. In later applications, breakpoints were identified by chromosome walking, a procedure that requires the availability of the genome sequence of either the species under study or that of a closely related species to design probes that are physically mapped by *in situ* hybridization on polytene chromosomes^11,16–20^. These experimental procedures have allowed the identification and characterization of the breakpoints of three autosomal inversions of *D*. *melanogaster* ^14–16^ that were later also identified by computational methods^12^. They have also allowed the identification and characterization of the breakpoints of one autosomal inversion of *D*. *buzzatii* ^21^ and eight autosomal inversions of *D*. *subobscura* ^11,17–20^. In the latter species, the breakpoints characterization also revealed that most of the studied inversions (seven of eight) had originated through the staggered-breaks mechanism, with the duplicated fragment ranging in this case from 60 bp to ~7.8 kb.

*D*. *subobscura* exhibits a rich chromosomal polymorphism that affects its five large chromosomal elements (*i*.*e*., its four large autosomes, and the X or A chromosome). Geographical and temporal studies of this polymorphism point to the adaptive character of some chromosomal arrangements. Indeed, latitudinal clines were detected for chromosomal arrangements of the species five large elements in both the Old World and the newly colonized areas of the New World^22,23^. Moreover, temporal studies of population variation revealed concordant changes in the frequencies of chromosomal arrangements of the five elements and temperature^3,24^.

Here, we have focused on two inversions of the A chromosome of *D*. *subobscura* —A_2_ and A_1_. Inversion A_2_ is present both in the species ancestral distribution area and in the west coast of both North and South America. It is considered a warm-climate arrangement both because its frequency decreases with latitude in the three areas^23^ and because its frequency changes are concordant with temperature changes in long-term temporal studies^3^. Inversion A_1_ has also a widespread presence in the species ancestral distribution area where it presents an east-west cline^22^, but it has not been detected in the newly colonized areas possibly due to its rather low frequency in western European populations (*i*.*e*., in populations from which the colonizers might have stemmed^25–27^). Our identification and characterization of the A_2_ inversion breakpoints further supports the prevalent character of the staggered-breaks mechanism to originate inversions in *D*. *subobscura*, and it will facilitate subsequent work aimed to establish the genetic basis of its inversion polymorphism adaptive character. In contrast, we have been unable to cross any breakpoint of the A_1_ inversion by chromosome walking even though they have been both rather narrowly delimited. We discuss the limitations imposed by using the genome of a moderately related species to guide chromosomal walks for a chromosome that in the Drosophila genus exhibits the highest rate of evolution^28–30^.

## Results

### Identification and characterization of inversion A_2_ breakpoints

**Figure 1:**
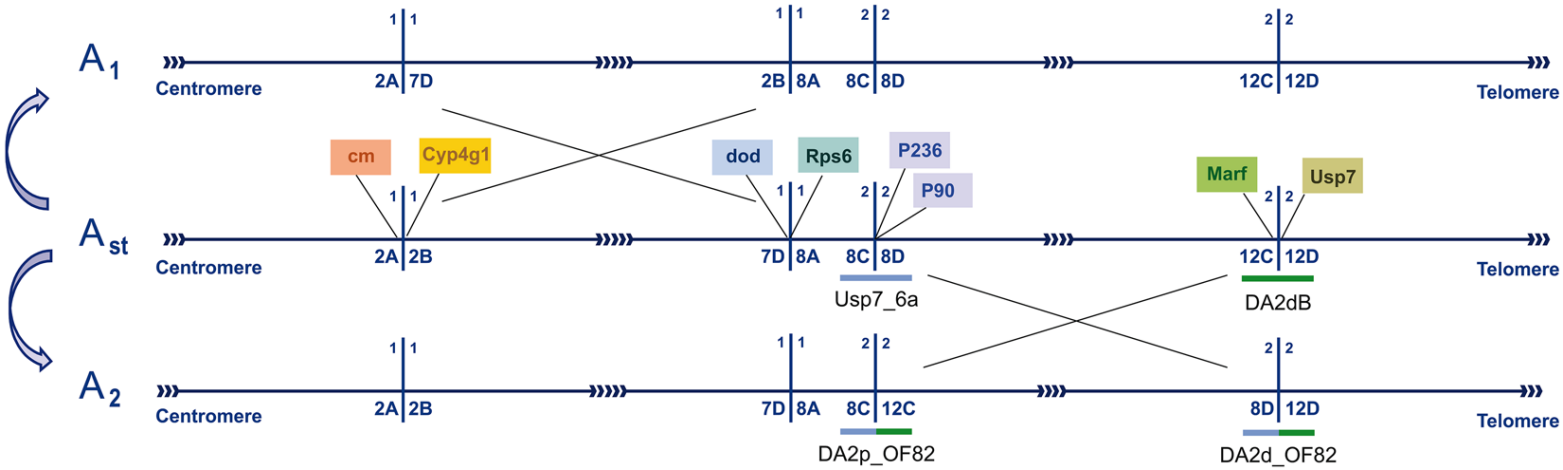
Schematic representation of the A chromosome regions of *Drosophila subobscura* affected by inversions A_2_ and A_1_. Horizontal lines connected by arrows represent the different chromosomal arrangements —the A_st_ arrangement in the center and the A_1_ and A_2_ arrangements above and below, respectively—, whereas vertical lines represent the different inversion breakpoints with indication of their location (section) on the Kunze-Mühl and Müller (1958) map. Pairs of crossed lines between arrangements indicate inverted regions. The location of the initial markers used for chromosomal walks are represented in colored boxes on the A_st_ arrangement. Short blue and green horizontal lines represent the fragments spanning the A_2_ inversion breakpoint regions with the name of the probes below.

The breakpoints of inversion A_2_ are located at sections 8C/8D and 12C/12D on the A_st_ Kunze-Mühl and Müller^31^ cytological map (Fig. 1). Four markers that had been previously mapped close to the inversion breakpoints were used to start chromosomal walks towards the breakpoints using the *D*. *pseudoobscura* and *D*. *melanogaster* genomes as a guide: markers P90 and P236 for the proximal breakpoint, and markers *Marf* and *Usp7* for the distal breakpoint^30,32^. In the *D*. *pseudoobscura* genome, markers P90, P236 and *Usp7* are rather closely located (Supplementary Fig. S1). This observation led us to design several probes in this region. We also designed probes flanking the *Marf* region in *D*. *pseudoobscura*. All probes were hybridized in both arrangements —A_st_ and A_2_— to obtain position information relative to each breakpoint of the inverted region.

For the proximal breakpoint, probes flanking marker P236 gave a single signal at section 8D whereas that flanking marker P90 did at section 8E/9A (Supplementary Fig. S1). As marker Usp7_4 mapped closer to the breakpoint than the rest of markers, four additional probes were designed in that direction. Two of these probes mapped at section 8D —inside of the inverted region— and the other two at section 8C —outside of the inverted region—, thus delimiting the breakpoint. Even though several probes were designed in the intervening region, only one of them —Usp7_6a— could be successfully amplified in the *ch cu* strain (Supplementary Fig. S1). When *in situ* hybridized on A_st_ and A_2_ chromosomes, this probe gave a single signal at section 8C/D on A_st_ chromosomes, and two distant signals at sections 8C next to 12C and 8D next to 12D on A_2_ chromosomes (Fig. 1 and Supplementary Fig. S2), indicating that this probe included the proximal breakpoint.

For the distal breakpoint, probes flanking the *Usp7* marker that had been designed in the P236-P90-*Usp7* region of *D*. *pseudoobscura* either mapped at section 10A or at section 12D like the *Usp7* marker did, but moving away from the breakpoint (Supplementary Fig. S1). Probes were also designed on both sides of *Marf* in the *D*. *pseudoobscura* genome (Supplementary Fig. S1). In *D*. *subobscura*, hybridization signals revealed that the probes mapping at the 12C section were moving away from the breakpoint whereas the remaining probes mapped at different regions of the A chromosome (Supplementary Fig. S1). As collinearity with the *D*. *pseudoobscura* and *D*. *melanogaster* genome sequences was lost in both the *Usp7* and *Marf* regions, we searched draft2 of the *D*. *subobscura* genome (Barcelona Subobscura Initiative [BSI]) and detected an ~21-kb long scaffold that included the *Marf* gene. Comparison of this scaffold with the *D*. *pseudoobscura* and *D*. *melanogaster* genomes revealed a new collinear region between both genomes, which allowed us to design three new probes that led to the identification of the breakpoint region and to design a final probe spanning it —probe DA2dB (Supplementary Fig. S1). This probe gave a single hybridization signal at section 12C/D on A_st_ chromosomes and two distant signals at sections 12C next to 8C and 12D next to 8D, respectively, on A_2_ chromosomes (Supplementary Fig. S2).

Upon identification of the breakpoint regions in A_st_ chromosomes, the fragments spanning these breakpoints in A_2_ chromosomes could be amplified with the corresponding combinations of oligonucleotides (Fig. 1). Their *in situ* hybridization on A_st_ chromosomes gave two clear signals at the breakpoints sections according to the Kunze-Mühl and Müller^31^ cytological map (Supplementary Fig. S3). These results confirmed that the amplified fragments included the corresponding breakpoints in A_2_ chromosomes. However, these fragments also gave two signals on A_2_ chromosomes (see below) and multiple signals in centromeric regions.

**Figure 2:**
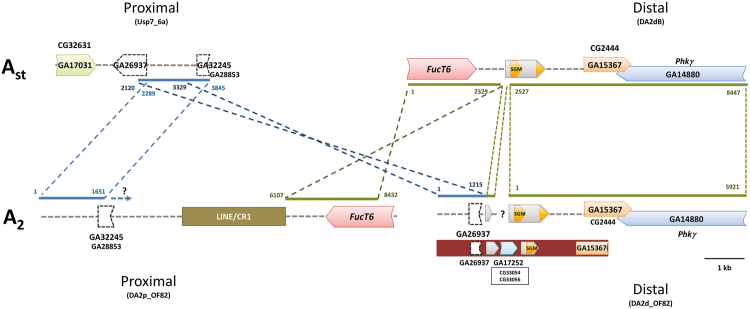
Schematic representation of inversion A_2_ breakpoint regions in chromosomal arrangements A_st_ and A_2_. In each sequenced fragment, boxes represent coding regions and transposable elements whereas discontinuous lines represent intergenic regions. Upper images refer to the breakpoint fragments sequenced in the *ch cu* strain whereas lower images refer to those sequenced in the OF82 and OF79 strains. Thin discontinuous lines between arrangements indicate the limits and orientation of homologous regions, with numbers indicating their location in the sequenced fragments. For each coding region, the name of its ortholog in either *D*. *melanogaster* or *D*. *pseudoobscura* is given. ?, missing information.

Fragments spanning the proximal and distal breakpoint regions were sequenced in the *ch cu* (A_st_) and OF82 (A_2_) strains, respectively (Fig. 2). The distal breakpoint fragment could not be completely sequenced in the OF82 strain, which led us to sequence it in a second A_2_ (OF79) strain. The detailed analysis of these fragments in non-inverted (A_st_) and inverted (A_2_) chromosomes led to their annotation, whereas their pairwise comparison allowed us to delimit the breakpoints (Fig. 2).

For the proximal A_st_ breakpoint region, the ~3.8-kb long sequenced fragment (Usp7_6a) contains the GA26937 gene flanked by part of the CG32631 and GA32245 genes (Fig. 2). For the distal A_st_ breakpoint region, the ~8.4-kb long sequenced fragment (DA2dB) contains the CG2444 gene and part of the *FucT6* and *Phkγ* genes. In A_2_ chromosomes, part of the intergenic region between genes GA26937 and GA32245 is present in both the proximal and distal inversion breakpoints (Fig. 2), indicating that inversion A_2_ originated through the staggered-breaks mechanism. Even though the breakpoints could be accurately identified through the pairwise comparison of A_st_ and A_2_ breakpoint regions, the extent of the duplicated fragment could not be established given the length of the fragment that could be successfully amplified and sequenced in the *ch cu* strain, even though it has a minimal length of ~1.7 kb (Fig. 2).

### Identification of inversion A_1_ breakpoints

The breakpoints of inversion A_**1**_ are located at sections 2A/B and 7D/8A on the A_st_ Kunze-Mühl and Müller^31^ cytological map (Fig. 1). Four markers previously mapped near the inversion breakpoints were used for chromosome walking using the *D*. *pseudoobscura* and *D*. *melanogaster* genomes as a guide: markers *cm* and *Cyp4g1* for the proximal breakpoint, and markers *dod* and *RpS6* for the distal breakpoint^30^.

For the proximal breakpoint, one or more probes were designed on both sides of the *D*. *pseudoobscura* homologs of markers *cm* and *Cyp4g1*. In both attempts to walk, *in situ* hybridization revealed that probes on one side of the focal probe mapped at other parts of the X chromosome (Supplementary Fig. S4), and probes on the other side moved away from the corresponding breakpoint. We therefore searched draft2 of the *D*. *subobscura* genome (BSI) and detected two scaffolds that contained the *cm* and *Cyp4g1* genes, respectively. Comparison of these scaffolds with the *D*. *pseudoobscura* genome revealed an ~450-kb long region, each of which ends was collinear with one of the *D*. *subobscura* scaffolds (Supplementary Fig. S4). New probes could be therefore designed across this fragment and their *in situ* hybridization revealed that we were moving towards the breakpoint from both ends, being probes cmR5 and DA1p_sc36a the closest to each side of the breakpoint (Supplementary Fig. S4). Even though these probes were only ~30 kb apart in the *D*. *pseudoobscura* genome, our efforts to design a probe spanning the breakpoint were unsuccessful because this intervening fragment does not exhibit any homology with the *D*. *subobscura* genome draft. However, the partial superposition of markers cmR5 and DA1p_sc36a signals on A_st_ chromosomes —when used as probes for double-color fluorescent *in situ* hybridization (FISH; Supplementary Fig. S5)— clearly indicates that these markers narrowly delimit inversion A_1_ proximal breakpoint.

For the distal breakpoint, one or more probes were designed on both sides of the *D*. *pseudoobscura* homologs of markers *dod* and *RpS6* (Supplementary Fig. S4). For the *dod* region, *in situ* hybridization revealed that the internal probe DA1d_Evi5 moved closer to the breakpoint whereas other probes mapped at different regions of the A chromosome (Supplementary Fig. S4). For the *RpS6* region, *in situ* hybridization also revealed that probe RpS6Yp3_12a mapped closer to the breakpoint than the focal probe (Supplementary Fig. S4). The collinearity breaks of *D*. *subobscura* relative to the *D*. *pseudoobcura* and *D*. *melanogaster* genomes detected for both the *dod* and *RpS6* regions, and the negative results obtained when searching draft2 of the *D*. *subobscura* genome did not allow us to walk further to cross the breakpoint. Similarly to the A_1_ proximal breakpoint, this inversion distal breakpoint can also be considered rather narrowly delimited, in this case by markers DA1d_Evi5 and RpS6Yp3_12a as revealed by double-color FISH (Supplementary Fig. S5).

## Discussion

In Dipteran genera such as Drosophila and Anopheles, classical cytological studies revealed that many species harbor chromosomal polymorphism resulting from paracentric inversions. These studies also revealed that the number of polymorphic inversions varies among species as well as among chromosomal elements. In most polymorphic species of the Drosophila genus, polymorphism affects one or more of its large autosomal elements, ranging from multiple and complex rearrangements in a single chromosomal element in *D*. *pseudoobscura* (Muller’s C element) to chromosomal arrangements resulting from one or more inversions in the four large autosomal elements in *D*. *melanogaster* and *D*. *subobscura* ^33^. Concerning the X chromosome, most species are either monomorphic or exhibit only a complexly rearranged X chromosome associated to sex-ratio distortion, as it is the case in *D*. *pseudoobscura* ^33^. Other species such as *D*. *melanogaster* and *D*. *subobscura* exhibit simple polymorphic inversions in the X chromosome, two endemic inversions in the former species —one of them associated to a sex-ratio distorter^13^— and two widely distributed inversions in the latter species that also exhibits complex arrangements including one associated to sex-ratio distortion^22^. The generalized paucity of X-chromosome inversions segregating at some frequency in natural populations of multiple species stands in contrast with this element exhibiting the highest rate of chromosomal evolution in the Drosophila genus^28–30^.

In *D*. *melanogaster*, the set of eight inversions with breakpoints molecularly characterized^13–16^ included both autosomal and X-chromosome inversions whereas the *D*. *subobscura* set of also eight inversions only included autosomal inversions^11,17–20^. This characterization revealed that most of them originated by the SSB mechanism. The present characterization of the A_2_ inversion breakpoints revealed that a fragment present only at the proximal breakpoint in A_st_ chromosomes was present at both breakpoints of A_2_ chromosomes, an indication that it was duplicated during the inversion process. This observation would support that this X-chromosome inversion had also originated by the staggered-breaks mechanism, even though the extent of the duplicated fragment could only be approximately established (≥1.7 kb). It should be noted that the distal breakpoint could be completely sequenced in only one of the two A_2_ strains, possibly due to changes accumulated after the inversion origin. Concerning our efforts to identify the A_1_ inversion breakpoints, we were not able to identify at the nucleotide level any of its breakpoints, but their location was in both cases rather narrowly delimited as revealed by double-color FISH.

Computational methods developed for the *de novo* identification of polymorphic inversion breakpoints using next-generation sequenced (NGS) genomes require a high-quality genome of the species under study, as they are based on the mapping of the next-generation paired-end or mate paired reads^12,34,35^. These methods were initially developed and mainly applied to samples from *Drosophila melanogaster* and *Homo sapiens*, which are two model species with high quality reference genomes (dm6; hg38). Even in these species, it is important to discriminate true from false positive predictions, which can be accomplished by gathering available population-level information as well as by designing assays for the experimental validation of predicted inversions^12,34,36^. In Drosophila, like in many other genera, the number of species with sequenced genomes is steadily increasing [from 12 in 2007 to the 22 nowadays accessible in flybase (http://flybase.org/)], even though the new genome assemblies generally exhibit a rather low contiguity. Regions rich in transposable elements and other repetitive sequences are often over-represented in unassigned contigs as well as at the ends of large scaffolds, which contributes to increased discontinuity. Inversion breakpoints are known to accumulate transposable elements as a result of their decreased rate of excision, which might therefore limit old inversion breakpoints identification by computational methods. The above-mentioned characteristics of genomes and of genome assemblies would therefore preclude the successful use of the developed computational methods to identify many inversion breakpoints, particularly in species rich in repetitive elements.

Concerning experimental methods to identify an inversion breakpoints (*e*.*g*., by chromosomal walks) in species without a high-continuity reference genome using as a reference a good-quality related species genome, limitations stem from the rate of evolution by paracentric inversions of the chromosomal element under study since the two species diverged. Indeed, the collinearity break imposed by an interspecific inversion can only be overcome by the availability of either a second reference species genome exhibiting collinearity with the study species at that region, or of some scaffolds of the species under study covering the region where collinearity was lost relative to the reference species genome. We have encountered different degrees of difficulty in identifying inversion breakpoints in the non-inverted chromosome of *D*. *subobscura* by chromosome walking using as references both the *D*. *pseudoobscura* and *D*. *melanogaster* genomes and also the initial and highly discontinuous drafts of the *D*. *subobscura* genome^11,17–20^. Indeed, we have been able to cross the inversion breakpoints in the eight autosomal inversions with breakpoints previously characterized, even though we were unable to sequence to completion one of the inverted breakpoint regions^19^. In contrast, we have been able to cross the breakpoints of only one of the two X-chromosome inversions here characterized (inversion A_2_). In the second X-chromosome inversion (A_1_), we have approached each of its two breakpoints from both sides, but we have been unable to cross any of them. Moreover, even though breakpoints have been narrowed down to a presumably small fragment in both non-inverted and inverted chromosomes, we have not been able to amplify the corresponding regions.

In summary, the identification and subsequent characterization of the breakpoints of two widely distributed X-chromosome inversions of *D*. *subobscura* —inversions A_2_ and A_1_— has only been successful for the A_2_ inversion. The duplication detected at both breakpoints would be consistent with it having originated by the staggered-breaks mechanism, as also did seven of the eight autosomal inversions with breakpoints characterized. The breakpoints of the A_1_ inversion could only be rather narrowly delimited. Our failure to cross the latter inversion breakpoints by chromosome walking highlights the limitations of this experimental approach when it relies on the genome sequence of a moderately distant species, particularly when the affected element exhibits a high rate of chromosomal evolution.

## Materials and Methods

### Molecular breakpoint characterization

Four homokaryotypic strains of *D*. *subobscura* were used to molecularly identify the breakpoints of inversions A_2_ and A_1_: A_st_ (*ch cu*), A_2_ (OF82 and OF79) and A_1_ (OF74). The OF strains are isogenic strains obtained as described in Puerma *et al*.^18^.

Molecular markers previously mapped in the vicinity of each inversion breakpoints were used as starting points of the corresponding chromosomal walks. Successive sets of probes were physically mapped on polytene chromosomes by *in situ* hybridization, which allowed walking towards each breakpoint until its eventual identification. Probes design strategy was based on the collinearity between the *D*. *pseudoobscura* and *D*. *melanogaster* genomes with that of *D*. *subobscura*, but oligonucleotides design for probes amplification was based on the draft2 of the *D*. *subobscura* genome sequence (BSI) as described in Puerma *et al*.^18^. Probes were amplified by PCR using TaKaRa DNA polymerase (Takara Bio Inc) and genomic DNA from the *ch cu* strain [using the Puregen Cell kit B (Qiagen)].

Probes were Biotin-16-dUTP (Roche) labeled and *in situ* hybridized on polytene chromosomes of either the *ch cu* and OF82 strains (A_2_ inversion) or the *ch cu* and OF74 strains (A_1_ inversion). However, the final probes of the A_1_ chromosomal walks were either Biotin-16-dUTP (Roche) or Digoxigenin-11-dUTP (Roche) labeled to carry out dual-color FISH on the same strains. For fluorescence detection, either Dylight 549 streptavidin or Dylight 488 anti-digoxigenin (Vector Laboratories Inc.) were used. Polytene chromosome visualization was performed with a Vectashield Mounting Media (Vector Laboratoires Inc.) and DAPI solution. Hybridization signals were subsequently located on the cytological map of *D*. *subobscura* ^31^. All steps of the *in situ* hybridization procedure were performed as described in Montgomery *et al*.^37^ with minor modifications, and adapted in the case of dual-color FISH. Digital images at a 400 magnification were obtained using either a Leica DFC290 camera mounted on a phase contrast Axioskop 2 Zeiss microscope or an inverted fluorescence microscope and the Leica Application Suite (LAS) program. The latter images were subsequently processed using the ImageJ 1.50 g program^38^.

### Breakpoint Sequence analysis

Fragments spanning the breakpoints were PCR amplified using DNA from both non-inverted and inverted strains using TaKaRa DNA polymerase (Takara Bio Inc) and oligonucleotides anchored at each breakpoint flanking regions. The amplified fragments were sequenced using primer walking whenever necessary. Amplicons were purified with MultiScreen PCR plates (Millipore) prior to their sequencing with the ABI PRISM version 3.2 cycle sequencing kit. Sequencing products separated on an ABI PRISM 3730 sequencer. All sequences were obtained on both strands and assembled using the DNASTAR package^39^. Sequences newly obtained have been deposited in the EMBL/GenBank Data Libraries under accession numbers LT963531-LT963536.

Comparison of the breakpoint regions with the *D*. *pseudoobscura* genome (FlyBase; http://flybase.org/) using BLAST tools allowed their annotation with genes. Breakpoint regions were also analyzed with RepeatMasker (http://repeatmasker.org/) to detect transposable elements and other repetitive sequences. The Align Sequences Nucleotide BLAST utility at the NCBI webpage was used to finely establish each breakpoint and to determine putative duplications resulting from the inversion process.

## Electronic supplementary material

Supplementary information
