# Supplementary material for: Inversion evolutionary rates might limit the experimental identification of inversion breakpoints in non-model species
Source: Sci Rep. 2017 Dec 8;7:17281. doi: 10.1038/s41598-017-17650-1 (PMC5722822; doi:10.1038/s41598-017-17650-1)
Supplement: Supplementary file 1 — Supplementary information [file 41598_2017_17650_MOESM1_ESM.pdf]

Supplementary Information

Inversion evolutionary rates might limit the experimental identification of inversion breakpoints in non-model species

Eva Puerma, Dorcas J. Orengo and Montserrat Aguadé*

Departament de Genètica, Microbiologia i Estadística, Facultat de Biologia and Institut de Recerca de la Biodiversitat (IRBio), Universitat de Barcelona, Barcelona, Spain

* Author for Correspondence

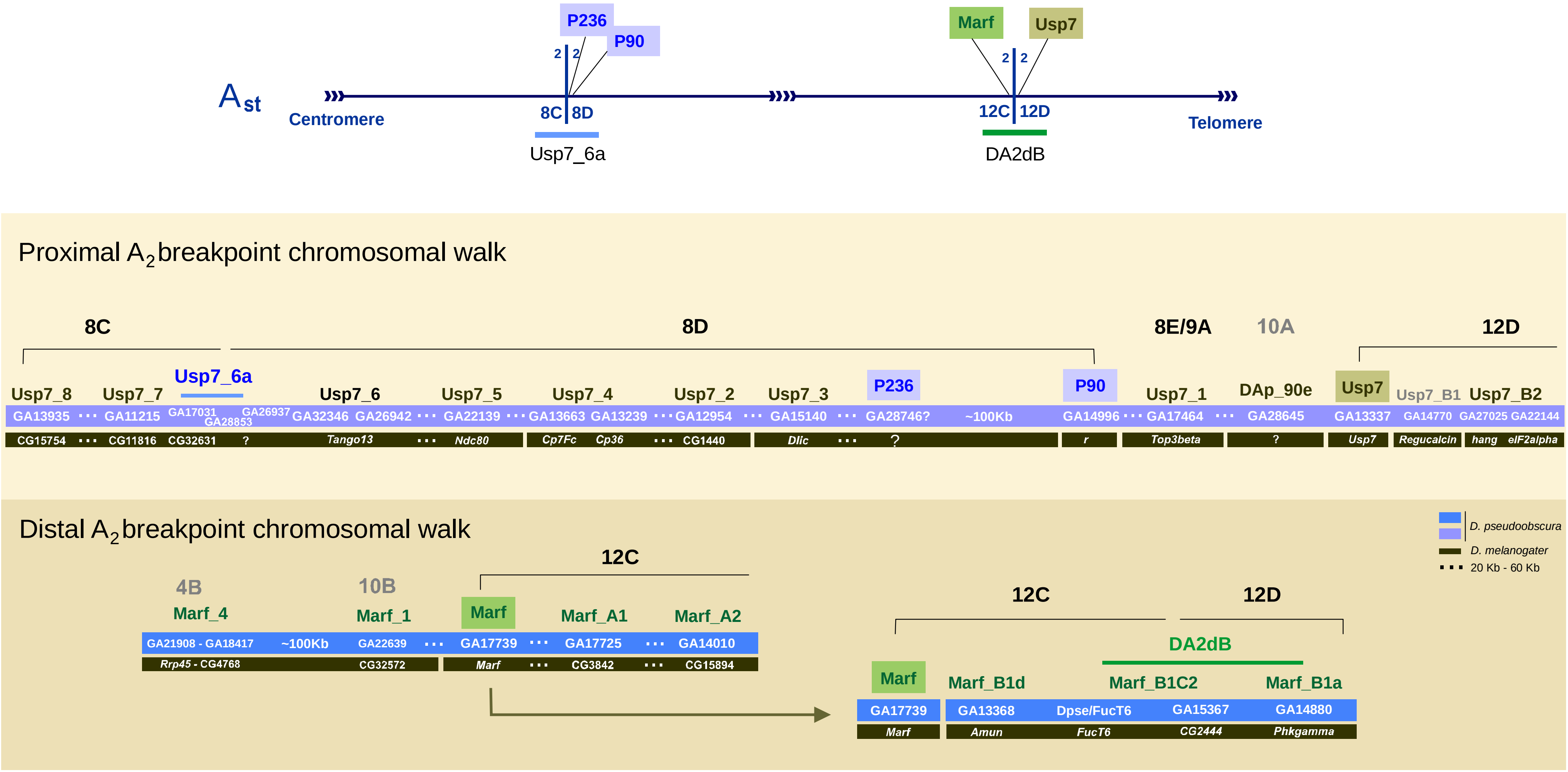

Supplementary Figure S1. Chromosomal walks performed to identify inversion A_2 breakpoints. Schematic representation of inversion A_2 breakpoints on A_{st} chromosomes including the location of the initial markers used (upper part), and detailed schematic representation of the chromosomal walks performed to identify inversion A_2 breakpoints on A_{st} chromosomes (lower part). Colored bars (not at scale) represent conserved syntenic blocks between the *D. pseudoobscura* and *D. melanogaster* genomes. Probes names and their location (section) on the Kunze-Mühl and Müller (1958) map of *D. subobscura* are indicated above those bars. Probes spanning each breakpoint are underlined and highlighted in blue and green for the proximal and distal breakpoints, respectively. Names within bars indicate the corresponding orthologous genes that are contiguous except when separated by small squares.

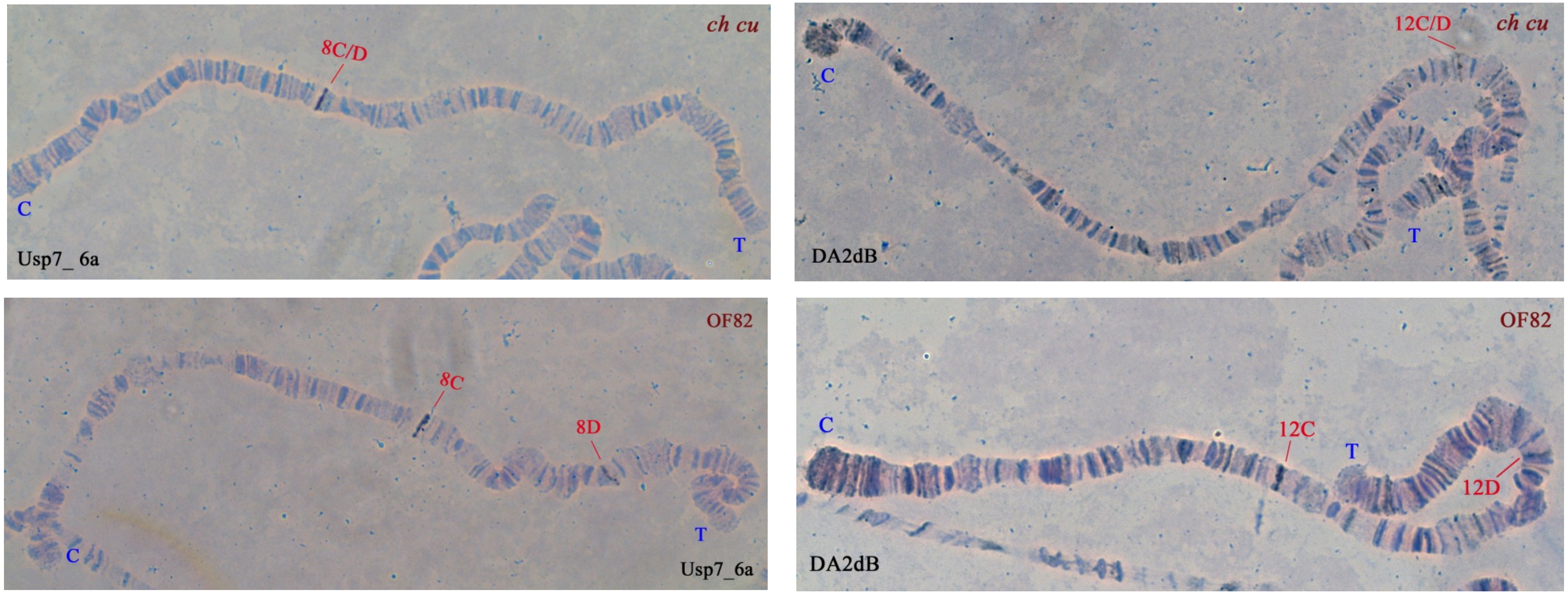

Supplementary Figure S2. *In situ* hybridization results using probes spanning the A_2 inversion breakpoints in the *ch cu* (A_{st}) strain. Results of the *in situ* hybridizations performed on the *ch cu* (A_{st}) and OF82 (A_2) strains using as probes the fragments spanning inversion A_2 breakpoints amplified in the *ch cu* strain (Usp6a and DA2dB for the proximal and distal breakpoints, respectively). Hybridization signals are marked with a red line and their cytological location is also indicated in red. T, telomere; C, centromere.

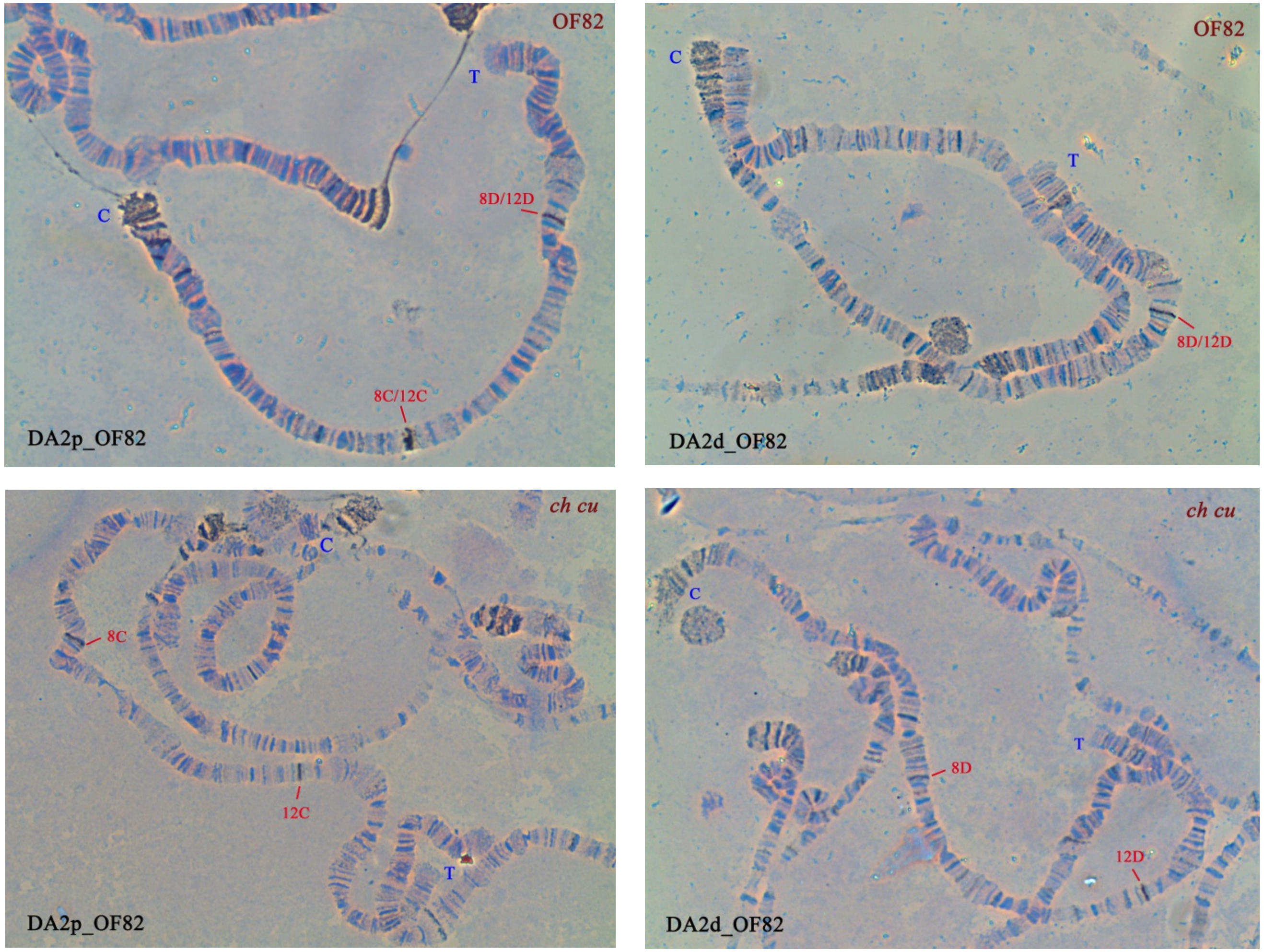

Supplementary Figure S3. *In situ* hybridization results using probes spanning the A_2 inversion breakpoints in the OF82 (A_2) strain. Results of the *in situ* hybridizations performed on the OF82 (A_2) strain using as probes the fragments spanning inversion A_2 breakpoints amplified in the OF82 strain (DA2p_OF82 and DA2d_OF82 for the proximal and distal breakpoints, respectively). Hybridization signals are marked with a red line and their cytological location is also indicated in red. T, telomere; C, centromere.

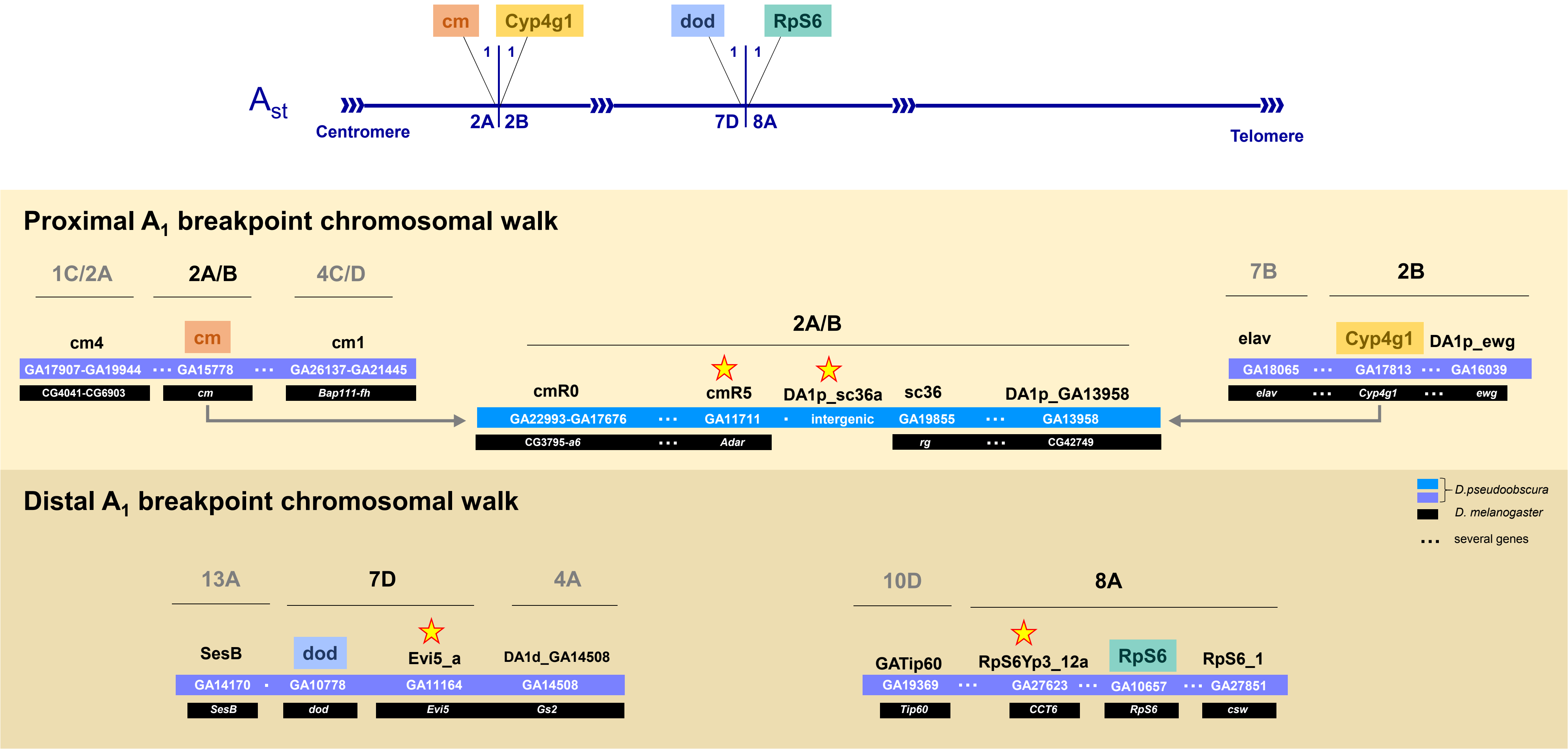

Supplementary Figure S4. Chromosomal walks performed to identify inversion A_1 breakpoints. Schematic representation of inversion A_1 breakpoints on A_{st} chromosomes including the location of the initial markers used (upper part), and detailed schematic representation of the chromosomal walks performed to identify inversion A_1 breakpoints on A_{st} chromosomes (lower part). Colored bars (not at scale) represent conserved syntenic blocks between the *D. pseudoobscura* and *D. melanogaster* genomes. Probes names and their location (section) on the Kunze-Mühl and Müller (1958) map of *D. subobscura* are indicated above those bars. Names within bars indicate the corresponding orthologous genes that are contiguous except when separated by small squares. Probes that delimit each breakpoint are highlighted with stars.

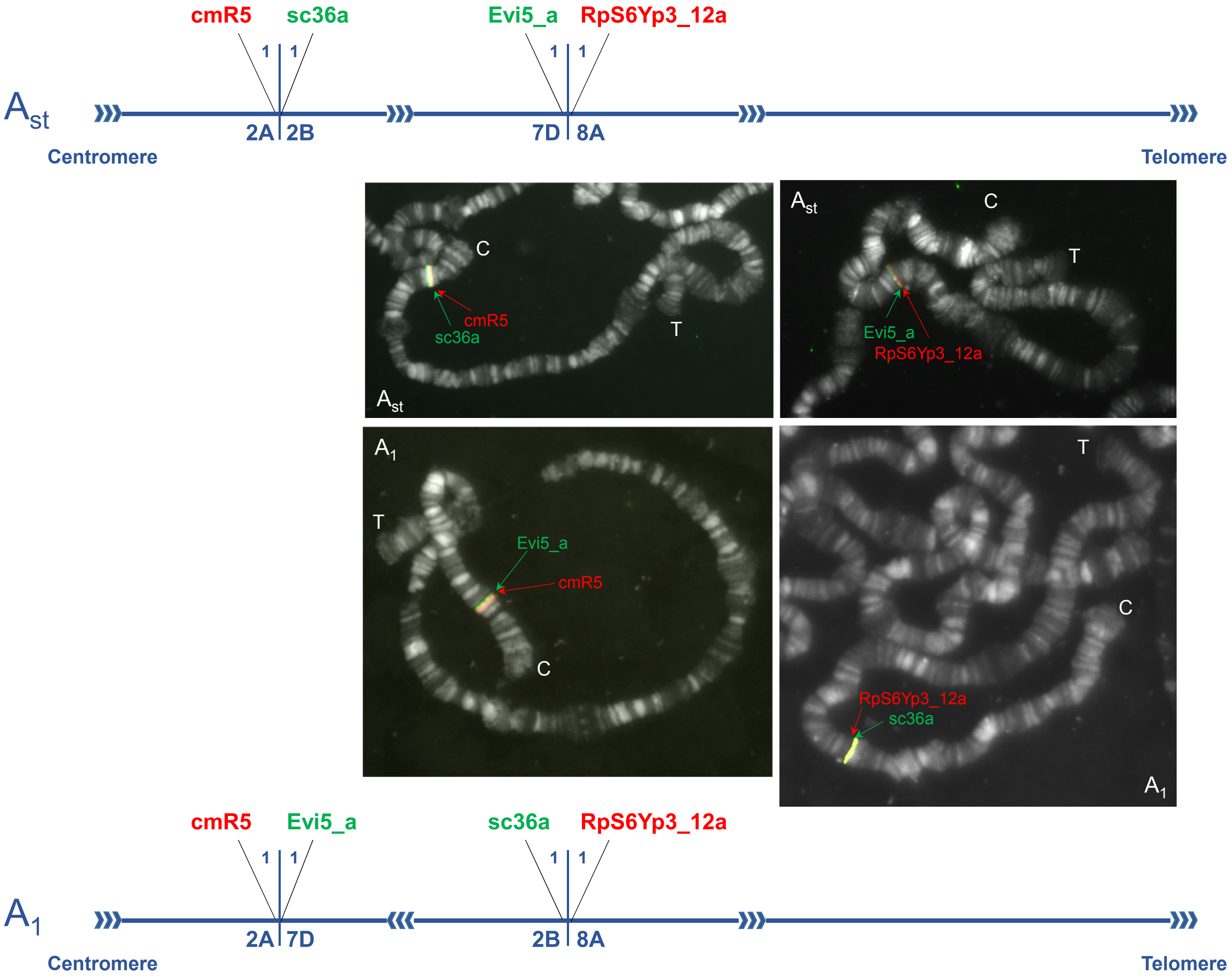

Supplementary Figure S5. Delimitation of inversion A_1 breakpoints. Schematic representation of inversion A_1 breakpoints and their delimiting probes on A_{st} (upper part) and A_1 (lower part) chromosomes, and fluorescent double-color in situ hybridization (FISH) results using these probes in the *ch cu* (A_{st}) and OF74 (A_1) strains (central part). Probes names are colored according to the fluorochrome used. Yellow signals correspond to the overlap of red and green signals with similar intensities. T, telomere; C, centromere.
